# Impact of COVID-19 on the health and psychosocial status of vulnerable older adults: study protocol for an observational study

**DOI:** 10.1186/s12889-020-09900-1

**Published:** 2020-11-30

**Authors:** Gary Cheung, Claudia Rivera-Rodriguez, Adrian Martinez-Ruiz, Etuini Ma’u, Brigid Ryan, Vanessa Burholt, Ange Bissielo, Brigette Meehan

**Affiliations:** 1 Department of Psychological Medicine, The University of Auckland, Private Bag 92019, Auckland Mail Centre, Auckland, 1142 New Zealand; 2 Brain Research New Zealand - Rangahau Roro Aotearoa, The University of Auckland, Auckland, New Zealand; 3 Department of Statistics, The University of Auckland, Auckland, New Zealand; 4 Department of Demographic Epidemiology and Social Determinants, National Institute of Geriatrics of Mexico, Mexico City, Mexico; 5 Department of Anatomy and Medical Imaging, The University of Auckland, Auckland, New Zealand; 6 School of Nursing / School of Population Health, The University of Auckland, Auckland, UK; 7 Centre for Innovative Ageing, College of Human and Health Sciences, Swansea University, Wales, New Zealand; 8 interRAI Services, Technical Advisory Services (TAS) Limited, Wellington, UK

**Keywords:** Pandemic, Older adults, Health, Mental health, interRAI

## Abstract

**Background:**

Many countries around the world have adopted social distancing as one of the public health measures to reduce COVID-19 transmissions in the community. Such measures could have negative effects on the mental health of the population. The aims of this study are to (1) track the impact of COVID-19 on self-reported mood, self-rated health, other health and psychosocial indicators, and health services utilization of people who have an interRAI assessment during the first year of COVID-19; (2) compare these indicators with the same indicators in people who had an interRAI assessment in the year before COVID-19; and (3) report these indicators publicly as soon as data analysis is completed every 3 months.

**Methods:**

interRAI COVID-19 Study (iCoS) is an observational study on routinely collected national data using the interRAI Home Care and Contact Assessment, which are standardized geriatric assessment tools mandated for all people assessed for publicly funded home support services and aged residential care in New Zealand. Based on the 2018/19 figures, we estimated there are 36,000 interRAI assessments per annum. We will compare the four post-lockdown quarters (from 25th March 2020) with the respective pre-lockdown quarters. The primary outcomes are self-reported mood (feeling sad, depressed or hopeless: 0 = no, 1 = yes) and self-rated health (0 = excellent, 1 = good, 2 = fair, 3 = poor). We will also analyze sociodemographics, other secondary health and psychosocial indicators, and health services utilization. Descriptive statistics will be conducted for primary outcomes and other indicators for each of the eight quarters. We will compare the quarters using regression models adjusted for demographic characteristics using weights or additional variables. Key health and psychosocial indicators will be reported publicly as soon as data analysis is completed for each quarter in the 12-month post-lockdown period by using a data visualization tool.

**Discussion:**

This rapid translation of routinely collected national interRAI data will provide a means to monitor the health and psychosocial well-being of vulnerable older New Zealanders. Insights from this study can be shared with other countries that use interRAI and prepare health and social services for similar epidemics/pandemics in the future.

## Background

As in many countries around the world, New Zealand went into lockdown at the end of March 2020 in an attempt to reduce the spread of COVID-19 infection in our communities. The sudden adoption of lockdown measures changed our social behavior and support networks overnight. Older adults are the most at-risk group for COVID-19 because its mortality risk increases with age, particularly in those with chronic conditions [1]. In New Zealand, as of the end of September 2020, 25 people had died of COVID-19; most of them are older adults. The lockdown measure urged adults aged 70 years old and above to avoid social contact and stay home. Such drastic measures can have many direct and indirect effects on the health and psychosocial well-being in the elderly. For example, older adults who were previously mobile but are now housebound are at risk of developing frailty [2]. Furthermore, the World Health Organization suggested that older adults in isolation might become more anxious, angry, stressed, agitated and withdrawn during the COVID-19 pandemic outbreak or while in isolation [3].

Some older adults in self-isolation can also become lonely if they live alone and/or cannot access their usual community programmes and support [1, 4]. Loneliness is a serious public health concern. It is a well-recognized risk factor for premature mortality, and poor physical and psychological wellbeing [5–8]. For example, previous studies have demonstrated that loneliness in older age is associated with reduced quality of life [9], higher rates of adverse health outcomes and higher mortality rates [6]. Moreover, social isolation possesses a premature mortality risk similar to the risk associated with maintaining an unhealthy diet, lack of physical activity and smoking [6]. It has been suggested that the impact of even short-term social distancing measures merits further study in populations where there is an existing high rate of loneliness and isolation [10].

Loneliness is relatively common in our community. Two recent studies found that one in five older New Zealanders assessed with the interRAI Home Care (interRAI-HC) assessment reported feelings of loneliness [11, 12], which is nearly twice the rate (11%) in the general older population [13]. For those interRAI subjects who were lonely, 27% had significant depressive symptoms and more than half (56%) stated poor or fair self-rated health [11].

interRAI-HC is a standardised geriatric assessment tool developed by health researchers in over 30 countries. New Zealand is the first country in the world to implement a mandated interRAI-HC for all people assessed for publicly funded home support services and aged residential care. Health professionals in the 20 District Health Boards (DHBs) use interRAI-HC to help determine which level of support older adults living in the community need. There were 36,000 interRAI-HC assessments completed in 2018/19 [14], while 10 and 40% of all New Zealanders aged 65 years and 85 years respectively have had an interRAI-HC during the same period. interRAI-HC contains information on about 250 demographic, clinical and psychosocial factors, which can be used to support care planning, resource allocation, quality measurement, and outcome evaluation [15]. It takes up to one and a half hours to complete an interRAI-HC assessment face-to-face in a person’s home. In response to the COVID-19 pandemic, interRAI New Zealand advised DHBs that they could use a shorter version of assessment, interRAI Contact Assessment, in place of interRAI-HC, between 25th March to 30th June 2020. The shorter version can be completed in 20 min over the telephone and allowed the assessment of patients urgently, reliably and efficiently.

Older adults assessed for home support services and aged residential care typically have physical illness and/or functional impairment [16]. Functional limitation is one of the main aetiological factors for loneliness in older adults [17–19]. Given the pre-existing high rates of loneliness, depression and poor/fair self-rated health in the interRAI population [11], we hypothesise that this population is at-risk of experiencing further decline in their health and psychosocial well-being as a result of the COVID-19 pandemic and self-isolation. The aims of this study are to (1) track the impact of COVID-19 on self-reported mood, self-rated health, other health and psychosocial indicators, and health services utilization of people who have an interRAI assessment during the first year of COVID-19; (2) compare these indicators with the same indicators in people who had an interRAI assessment in the year before COVID-19; and (3) report these indicators publicly as soon as data analysis is completed every 3 months.

## Methods/design

The interRAI COVID-19 Study (iCoS) is an observational longitudinal study of routinely collected national health information.

### interRAI data collection and data access

interRAI data are collected by trained interRAI assessors working in DHBs and approved community agencies that provide home support services. interRAI assessments are usually completed using a laptop computer, providing immediate outcomes at the time of assessment. There is a national competency framework that supports interRAI assessment and provides quality assurance for interRAI assessment. interRAI assessors must be clinically registered and signed off as competent to use the instruments by interRAI New Zealand. interRAI assessment competency is achieved by participating in training that involves a mixture of knowledge and skills, including completing real life assessments, passing an evaluation and achieving an acceptable quality review outcome. One of the competency standards is to ‘accurately record assessment information’ whereby an interRAI assessor uses multiple sources of information for example referral notes, person interview, observation, discussion with family or carers or health professionals to gain accurate information. The item domains of the interRAI Home Care version have been shown to have good inter-rater reliability [20].

**Fig. 1:**
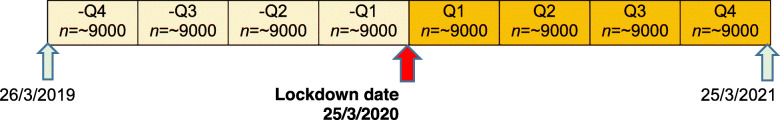
interRAI data collection and analysis timeline

interRAI data are entered in real-time to the Technical Advisory Services (TAS) data warehouse. Based on the 2018/19 figures, we estimate there are 9000 interRAI assessments per quarter (Fig. 1). We will compare the four post-lockdown quarters with the respective pre-lockdown quarters.

### Primary and secondary outcomes

interRAI data will be de-identified and analysed for each of the quarters shown in Fig. 1. The primary outcomes are self-reported mood (feeling sad, depressed or hopeless, 0 = no, 1 = yes) and self-rated health (0 = excellent, 1 = good, 2 = fair, 3 = poor). We will also analyse sociodemographics, other secondary health and psychosocial indicators, and health services utilization. Table 1 shows a summary of these variables and the re-coding performed to allow some direct comparison between both versions of interRAI assessments (Home Care and Contact Assessment) in the pre- and post-lockdown periods.

**Table 1:** InterRAI Home Care and interRAI Contact Assessment sociodemographic variables, health and psychosocial indicators, and health services utilization and their re-coding

Variables	Categories in interRAI Home Care	Categories in interRAI Contact Assessment	Re-coding
**Sociodemographic factors**			
Age	Continuous variable	Continuous variable	Categorise continuous variable to 1 = age 40–64, 2 = age 65–79 and 3 = age 80+
Gender	▪ Female ▪ Male ▪ Indeterminate ▪ Unknown	▪ Female ▪ Male ▪ Indeterminate ▪ Unknown	No change
Ethnicity	▪ European not further defined ▪ NZ European ▪ Other European ▪ Māori ▪ Pacific peoples not further defined ▪ Samoan ▪ Cook Island Māori ▪ Tongan ▪ Niuean ▪ Tokelauan ▪ Fijian ▪ Other Pacific peoples ▪ Asian not further defined ▪ Southeast Asian ▪ Chinese ▪ Indian ▪ Other Asian ▪ Middle Eastern ▪ Latin American/ Hispanic ▪ African (or any group of African origin) ▪ Other ethnicity ▪ Don’t know ▪ Refused to answer ▪ Response unidentifiable ▪ Not stated	▪ European not further defined ▪ NZ European ▪ Other European ▪ Māori ▪ Pacific peoples not further defined ▪ Samoan ▪ Cook Island Māori ▪ Tongan ▪ Niuean ▪ Tokelauan ▪ Fijian ▪ Other Pacific peoples ▪ Asian not further defined ▪ Southeast Asian ▪ Chinese ▪ Indian ▪ Other Asian ▪ Middle Eastern ▪ Latin American/ Hispanic ▪ African (or any group of African origin) ▪ Other ethnicity ▪ Don’t know ▪ Refused to answer ▪ Response unidentifiable ▪ Not stated	Re-code to 1 = European, 2 = Maori, 3 = Pacific, 4 = Asian, 5 = MELAA (Middle Eastern Latin American and African), 6 = Other
Living arrangement	▪ Alone ▪ With spouse/partner only ▪ With spouse/partner and other(s) ▪ With child (not spouse/partner) ▪ With parent(s) or guardian(s) ▪ With sibling(s) ▪ With other relative(s) ▪ With non-relative(s)	▪ Alone ▪ With spouse/partner only ▪ With spouse/partner and other(s) ▪ With child (not spouse/partner) ▪ With parent(s) or guardian(s) ▪ With sibling(s) ▪ With other relative(s) ▪ With non-relative(s)	Re-code to 1 = Alone, 2 = With spouse/partner only, 3 = Other (With spouse/partner and other(s) **or** With child (not spouse/partner) **or** With parent(s) **or** guardian(s) **or** With sibling(s) **or** With other relative(s) **or** With non-relative(s))
Domicile code	Domicile code of usual living arrangement	Domicile code of usual living arrangement	Re-code to 1 = Main urban areas, 2 = Satellite urban areas, 3 = Independent urban areas, 4 = Rural areas with high urban influence, 5 = Rural areas with moderate urban influence, 6 = Rural areas with low urban influence, 7 = Highly rural/remote areas, 8 = Area outside urban/rural profile.
Residential status	▪ Private home/apartment/rented room ▪ Board and care ▪ Assisted living or semi-independent living ▪ Mental health residence—e.g., psychiatric group home ▪ Group home for persons with physical disability ▪ Setting for persons with intellectual disability ▪ Psychiatric hospital/unit ▪ Homeless (with or without shelter) ▪ Long-term care facility (nursing home) ▪ Rehabilitation hospital/unit ▪ Hospice facility/palliative care unit ▪ Acute care hospital ▪ Correctional facility ▪ Other	▪ Private home/apartment/rented room ▪ Board and care ▪ Assisted living or semi-independent living ▪ Mental health residence—e.g., psychiatric group home ▪ Group home for persons with physical disability ▪ Setting for persons with intellectual disability ▪ Psychiatric hospital/unit ▪ Homeless (with or without shelter) ▪ Long-term care facility (nursing home) ▪ Rehabilitation hospital/unit ▪ Hospice facility/palliative care unit ▪ Acute care hospital ▪ Correctional facility ▪ Other	Re-code to 1 = Private home/apartment/rented room, 2 = Homeless (with or without shelter), 3 = Other (Board and care **or** Assisted living or semi-independent living **or** Mental health residence—e.g., psychiatric group home **or** Group home for persons with physical disability **or** Setting for persons with intellectual disability **or** Psychiatric hospital/unit **or** Long-term care facility (nursing home) **or** Rehabilitation hospital/unit **or** Hospice facility/palliative care unit **or** Acute care hospital **or** Correctional facility **or** Other)
**Physical/Clinical factors**			
Self-reported health	▪ Excellent ▪ Good ▪ Fair ▪ Poor ▪ Could/would not respond	▪ Excellent ▪ Good ▪ Fair ▪ Poor ▪ Could/would not respond	No change
Cognitive skills for daily decision making	▪ Independent ▪ Modified independence ▪ Minimally impaired ▪ Moderately impaired ▪ Severely impaired ▪ No discernable consciousness, coma	▪ Independent ▪ Modified independent or any impairment	interRAI-HC Re-code to 1 = Independent, 2 = Not fully independent (Modified independence **or** Minimally impaired **or** Moderately impaired **or** Severely impaired **or** No discernable consciousness, coma) interRAI-CA 1 = Independent, 2 = Not fully independent (Modified independent or any impairment)
Change in decision-making (as compared to 90 days or since last assessment)	▪ Improved ▪ No change ▪ Decline ▪ Uncertain	▪ Improved ▪ No change ▪ Decline ▪ Uncertain	No change
Falls	▪ Falls: in last 30 days - No falls - 1 fall - ≥2 falls ▪ Falls: 31–90 days ago - No falls - 1 fall - ≥2 falls	▪ No fall in last 90 days ▪ 1 or more falls in the last 90 days	interRAI-HC Re-code to 1 = No fall in last 90 days (No falls in last 30 days **or** No falls 31–90 days ago), 2 = 1 or more falls in last 90 days (≥1 falls in last 30 days **or** ≥ 1 falls 31–90 days ago) interRAI-CA 1 = No fall in last 90 days, 2 = 1 or more falls in last 90 days
Decrease in amount of food or fluid usually consumed	▪ Yes ▪ No	▪ Yes ▪ No	No change
Weight loss of 5% in last 30 days or 10% in last 180 days	▪ Yes ▪ No	▪ Yes ▪ No	No change
ADL self-performance ▪ Bathing ▪ Personal hygiene ▪ Dressing lower body ▪ Locomotion	▪ Independent ▪ Independent, setup help only ▪ Supervision ▪ Limited assistance ▪ Extensive assistance ▪ Maximal assistance ▪ Total dependence ▪ Activity did not occur during entire period	▪ Independent ▪ Supervision or any physical assistance	interRAI-HC Re-code to 1 = Independent, 2 = Not fully independent (Independent, setup help only **or** Supervision **or** Limited assistance **or** Extensive assistance **or** Maximal assistance **or** Total dependence **or** Activity did not occur during entire period) interRAI-CA 1 = Independent, 2 = Not fully independent (supervision or any physical assistance)
Change in ADL status	▪ Improved ▪ No change ▪ Declined ▪ Uncertain	▪ Improved ▪ No change ▪ Declined ▪ Uncertain	No change
IADL capacity ▪ Meal preparation ▪ Ordinary housework ▪ Managing medications ▪ Stairs	▪ Independent ▪ Setup help only ▪ Supervision ▪ Limited assistance ▪ Extensive assistance ▪ Maximal assistance ▪ Total dependence	▪ Independent or set-up help only ▪ Supervision or any assistance during task	interRAI-HC Re-code to 1 = Independent **or** setup help only, 2 = Not independent (Supervision **or** Limited assistance **or** Extensive assistance **or** Maximal assistance **or** Total dependence) interRAI-CA 1 = Independent or set-up help only, 2 = Not independent (Supervision or any assistance during task)
**Psychological indicators**			
Self-reported mood	“In the last 3 days, how often have you felt sad, depressed or hopeless?” ▪ Not in the last 3 days ▪ Not in the last 3 days, but often feels that way ▪ In 1–2 of the last 3 days ▪ Daily in the last 3 days ▪ Could/would not respond	“In the last 3 days, have you felt sad, depressed or hopeless?” ▪ Yes ▪ No ▪ Could/would not respond	interRAI-HC Re-code to 1 = Feeling depressed in last 3 days (In 1–2 of the last 3 days **or** Daily in the last 3 days), 2 = Not feeling depressed in last 3 days (Not in the last 3 days **or** Not in the last 3 days, but often feels that way), 3 = Could/would not respond interRAI-CA 1 = Yes, 2 = No, 3 = Could/would not respond
**Social indicators**			
Primary informal helper expresses feelings of distress, anger, or depression	▪ Yes ▪ No	▪ Yes ▪ No	No change
Family or close friends report feeling overwhelmed by person’s illness	▪ Yes ▪ No	▪ Yes ▪ No	No change
Two key informal helpers (relationship to person) ▪ Helper 1 ▪ Helper 2	▪ Child or child-in-law ▪ Spouse ▪ Partner/significant other ▪ Parent/guardian ▪ Sibling ▪ Other relative or whanau ▪ Friend ▪ Neighbour ▪ No informal helper	▪ Child or child-in-law ▪ Spouse ▪ Partner/significant other ▪ Parent/guardian ▪ Sibling ▪ Other relative or whanau ▪ Friend ▪ Neighbour ▪ No informal helper	Re-code to 1 = Spouse **or** Partner/significant other, 2 = Other family (Child or child-in-law **or** Parent/guardian **or** Sibling **or** Other relative or whānau), 3 = Friend **or** Neighbour, 4 = No informal helper
Two key informal helpers (lives with person) ▪ Helper 1 ▪ Helper 2	▪ No ▪ Yes, 6 months or less ▪ Yes, more than 6 months ▪ No informal helper	▪ No ▪ Yes, 6 months or less ▪ Yes, more than 6 months ▪ No informal helper	No change
**Lifestyle factors**			
Daily tobacco use	▪ Yes ▪ Not in last 3 days, but is usually a daily smoker ▪ No	▪ Yes ▪ Not in last 3 days, but is usually a daily smoker ▪ No	Re-code to 1 = Smoker (Yes **or** Not in last 3 days, but is usually a daily smoker), 2 = Non-smoker
**Health services utilisation**			
Time since last hospital stay	▪ No hospitalisation within 90 days ▪ 31–90 days ago ▪ 15–30 days ago ▪ 8–14 days ago ▪ In the past 7 days ▪ Now in hospital	▪ No hospitalisation within 90 days ▪ 31–90 days ago ▪ 15–30 days ago ▪ 8–14 days ago ▪ In the past 7 days ▪ Now in hospital	Re-code to 1 = No hospitalisation within last 30 days (No hospitalisation within 90 days **or** 31–90 days ago), 2 = Hospitalisation within last 30 days (15–30 days ago **or** 8–14 days ago, 3 = In the past 7 days **or** Now in hospital)
Emergency department use (number of times in last 90 days)	Continuous variable	Continuous variable	No change

*ADL* Activities of daily living, *IADL* Instrumental activities of daily living, *interRAI-CA* interRAI Contact Assessment, *interRAI-HC* interRAI Home Care

### Statistical analysis

#### The primary outcomes analysis

Initially, we will conduct descriptive statistics for primary outcomes and other indicators for each of the eight quarters (−Q4, −Q3, −Q2, −Q1, Q1, Q2, Q3, Q4) in Fig. 1. For example, rates of depressed/hopeless mood and fair/poor self-rated health. This analysis will describe the study sample and the distribution of explanatory variables to inform multivariate analysis strategies. To have comparable groups, we will adjust for confounders or modifiers such as demographic characteristics and factors associated with the assessment process using weights or propensity score matching methods.

Next, we will compare the pre-lockdown quarters with the post-lockdown quarters (−Q4 vs Q1, −Q3 vs Q2, −Q2 vs Q3, −Q1 vs Q4) by using regression models to control for the effect of demographic variables, physical or clinical factors, social indicators, lifestyle factors, or time of assessment. These factors will be incorporated in the model to test for significance.

The impact of COVID-19 measures on self-reported depressed mood (yes/no) will be evaluated using multivariate logistic regression. Self-rated health (0 = excellent, 1 = good, 2 = fair, 3 = poor) is an ordinal outcome and will be evaluated using ordinal logistic regression. If propensity score matching methods are used, we would use conditional logistic regression approaches. A stepwise selection of predictors will be used for inclusion in the model. Significant factors will be allowed to stay in the final model at *p*-value of less or equals to 0.05. Estimation approaches used for the regression models mentioned will be mixed models and generalized estimating equations (GEE). These approaches enable us to incorporate repeated measures in the analysis.

The fit and the validity of the models will be done by evaluating the deviance via goodness of fit tests. Multicollinearity will be tested using the variance inflation factor (VIF), which assesses how much the variance of an estimated regression coefficient increases if predictors are correlated. A VIF between 5 and 10 indicates high correlation that may be problematic.

R Statistical Software will be used for all analyses.

#### Sub-group analysis

We will perform additional data analysis for the following at-risk subgroups: living alone, cognitive difficulty and the older age group (age 80+).

### Data reporting and visualization

We will develop and publish an interactive report of key health and psychosocial indicators. The report will be publicly available as soon as data analysis is completed for each quarter in the 12-month post-lockdown period. TAS has existing infrastructure using Microsoft Power BI Service to publish interactive reports and dashboards such as interRAI reports available online at https://www.interrai.co.nz/data-and-reporting/. Power BI is a group of software services, apps, and connectors that work together to transform unrelated sources of data into coherent, visually immersive, and interactive insights [21]. The objectives of this Power BI report are to (i) provide interactive descriptive analysis to monitor self-reported mood, self-rated health, other health and psychosocial indicators, and health services utilization of the interRAI population every 3 months in the first year of COVID-19, and (2) allow pre- and post- COVID-19 comparative analyses of selected indicators. The research team will work with a TAS web designer and data visualization specialist to report our results on the interRAI New Zealand website.

## Discussion

There has been a call for urgent action to mitigate the physical and mental health consequences of COVID-19 self-isolation in older adults [4]. The ‘flattening the curve’ approach adopted by the New Zealand Government, including strict border control and quarantine was successful in practically controlling community transmission of COVID-19. The COVID-19 alert system implemented in New Zealand (Level 2 to 4) advises high-risk people to remain at home (e.g. those over 70 or those with other existing medical conditions). However, by easing some of these measures, many parts of the world are experiencing a second or third wave of COVID-19. Without this rapid translation of routinely collected national interRAI data, the health and psychosocial well-being of vulnerable older New Zealanders might be left un-monitored. If COVID-19 indeed has an impact on their health and psychosocial well-being, the New Zealand public health and social services and non-government organizations can ramp up their resources to address and minimize these problems at a national and/or community level as soon as our quarterly interRAI indicators become available. These rapid and effective responses could prevent further deterioration of the health of older New Zealanders, and decrease the impact on New Zealand health services. For example, evidence-based interventions to maximize mobility in older adults in isolation have been suggested [2], along with the use of a range of digital technologies for social engagement and measures to facilitate physical activity and nutrition [4, 22].

Physical illness, social isolation, and loneliness are well-recognised risk factors for late-life suicidal behaviour [23], especially during pandemic outbreaks [24]. For example, the rates of suicide among older adults increased in 2003 during the Severe Acute Respiratory Syndrome (SARS) epidemic in Hong Kong [24]. The effects of it lasted for the following years, since it was found that the suicide rates did not return to pre-epidemic rates [25]. In New Zealand, suicide rates of older men aged 85 years and above between 2011 and 2019 were the second highest amongst all age groups [26]. The social isolation and loneliness to which older adults are exposed during the COVID-19 pandemic in New Zealand could have important implications in increasing the already high suicide rates. interRAI includes information on self-rated health and depressed mood, which are two of the indicators we will report on. Many of the evidence-based late-life suicide prevention programmes target better screening and treatment of depression [27].

This study is a collaboration between the University of Auckland, interRAI New Zealand and TAS. interRAI New Zealand and TAS have expertise in interRAI data retrieval, analysis and dynamic reporting including web designing and data visualisation. interRAI Contact Assessment, a shorter version of interRAI-HC, replaced interRAI-HC in the first 3 months of the Level 4 lockdown in New Zealand. As shown in Table 1, some health indicators are not routinely collected by the interRAI Contact Assessment and this will result in missing data in the first post-lockdown quarter. Self-report mood and self-rated health are chosen as the two primary outcomes because they are included in both versions of interRAI assessments, which allows some direct comparison in the pre- and post-lockdown periods.

This study will generate a large health dataset that could be used to test other hypotheses. There is also an opportunity to link this dataset to other administrative datasets such as the Ministry of Health’s National Minimum Dataset (NMDS) for public and private hospital discharge information and Mortality Dataset for underlying causes of all registered deaths. We would welcome collaboration with other researchers and make our data available upon request and ethics approval. For example, Māori researchers who are interested in analysing health data related to the indigenous people of New Zealand. Our team already has members with expertise in Pacific and Asian health research who can interpret aspects of ethnicity-level data. Finally, insights from this study can be shared with other countries that use interRAI and prepare health and social services for similar epidemics/pandemics in the future.

## Trial status

Protocol version 1, 7 August 2020. interRAI data collection occurs in real-time to the TAS data warehouse. Data analysis for the 12-month pre-lockdown period and quarter 1 post-lockdown commenced on 12 August 2020.

## Data Availability

De-identified data and material are available upon request and ethics approval.

## Abbreviations

COVID-19: Coronavirus Disease of 2019
DHB: District Health Board
interRAI: International Residential Assessment Instrument
interRAI-HC: International Residential Assessment Instrument-Home Care
SARS: Severe Acute Respiratory Syndrome
TAS: Technical Advisory Services
